# Remote versus in-person consultations for acute cystitis: antibiotic treatment and clinical outcomes, a retrospective cohort study

**DOI:** 10.3399/BJGPO.2025.0009

**Published:** 2025-09-24

**Authors:** Lars Emil Aga Haugom, Knut Erik Emberland, Ingrid Keilegavlen Rebnord, Guri Rørtveit, Knut Eirik Ringheim Eliassen

**Affiliations:** 1 Department of Global Public Health and Primary Care, University of Bergen, Bergen, Norway; 2 Norwegian Institute of Public Health, Oslo, Norway

**Keywords:** cystitis, remote consultation, general practice, general practitioners, primary healthcare

## Abstract

**Background:**

The COVID-19 pandemic brought a surge of remote consultations in Norwegian primary care with unknown implications for antibiotic treatment and outcomes of acute cystitis.

**Aim:**

To examine whether there were differences in antibiotic treatment or repeat contacts for acute cystitis between remote and in-person consultations.

**Design & setting:**

A retrospective cohort study was undertaken. For the 4-year period from 2018–2021, we linked individual registry data on all contacts for cystitis by women aged ≥16 years in general practice and out-of-hours (OOH) services in Norway with registry data on dispensed antibiotics.

**Method:**

Index consultations for cystitis episodes were identified when there had been no urinary tract infection-related contacts or antibiotics dispensed at least 2 weeks before the consultation. We compared index remote versus index in-person consultations by antibiotic treatment within 3 days and repeat contacts within 14 days. Remote consultations were defined as consultations by text, telephone, or video.

**Results:**

Remote consultations for acute cystitis increased markedly, from 0.5% of acute cystitis consultations in 2018 to 15.2% in 2021. Index remote consultations were associated with more second-line antibiotic treatment (adjusted relative risk [aRR] 1.04, 95% confidence interval [CI] = 1.02 to 1.06, *P*<0.001), and more repeat contacts (aRR 1.11, 95% CI = 1.09 to 1.12, *P*<0.001) than in-person consultations.

**Conclusion:**

For acute cystitis, index remote consultations are associated with more second-line antibiotic treatment and more repeat contacts than in-person consultations. The unique time-period of the COVID-19 pandemic and the regular GP scheme in Norwegian primary care must be considered when interpreting these findings.

## How this fits in

Remote consultations for infections have been reported to possibly lead to higher prescription rates of antibiotics. The COVID-19 pandemic led to a sustained surge of remote consultations for all causes, with unknown implications for the antibiotic treatment and clinical outcomes of acute cystitis. The Norwegian Directorate of Health has, as of 2025, announced an intention to create a public online GP service and by law mandate regular GPs to offer remote consultations. This study compares remote and in-person consultations for acute cystitis in women aged ≥16 years in Norwegian primary care and shows that remote consultations are associated with more second-line antibiotics and more repeat contacts.

## Introduction

### Definitions, epidemiology, and aetiology

Cystitis is the most common bacterial infection in women, and eight out of 10 patients with cystitis are female.^1,2^ Acute cystitis can be classified as uncomplicated when occurring in healthy non-pregnant hosts without either structural abnormalities or instrumentation of their urinary tract. All other forms of cystitis can be considered complicated.^1^ Aetiology is *Escherichia coli* bacteria in around 80% of cystitis cases.^1,3^

### Clinical outcomes of acute cystitis

Uncomplicated cystitis in women is by a large margin the most common form of acute cystitis and is likely to be resolved within a week, regardless of treatment.^1,4^ A registry study from Denmark found repeat antibiotic treatment in <10% of uncomplicated cystitis cases.^5^ The risk of progression from cystitis to pyelonephritis is low; a nationwide cohort study from Sweden found that fewer than 1% of uncomplicated cystitis episodes were followed by a contact for pyelonephritis within 30 days.^6^ Complicated cystitis is a comparatively much more heterogeneous group regarding classification and associated outcomes.^7^

### Context and form of contact with healthcare services during the COVID-19 pandemic

Remote consultations were introduced in the public Norwegian primary care in 2013 as a type of contact equivalent to in-person consultations, with equal rights for the GPs to claim fees and reimbursement.^8^ There are three modalities of remote consultations in Norwegian primary care. Text messages between patients and physicians were introduced first in 2013, then came video consultation in 2016, and lastly telephone introduced in March 2020.^8^ Norwegian reimbursement claims have historically not differentiated between these forms of remote consultations,^9^ but started to do so as of July 2025.^10^ Text messaging by web browser, requiring secure personal login, is probably the most common form of remote consultations and has been described as the patient’s preferred access route to their regular general practitioner (RGPs).^11,12^ Video consultations made up one-third of all remote consultations in the early period of the COVID-19 pandemic.^13^ Use of video consultations has since decreased markedly to the low single digit percentages of all consultations.^14^

In the years leading up to the COVID-19 pandemic in 2020, remote consultations altogether made up less than 1% of all consultations.^15^ The first national restrictions owing to the COVID pandemic in Norway were introduced on 12 March 2020.^16^ In the following weeks, remote consultations surged, peaking at over 40% of all consultations and have since leveled at~25% of all consultations annually.^17^ The Norwegian Directorate of Health has, as of 2025, announced an intention to create a public online GP service and by law mandate RGPs to offer remote consultations.^18^

Internationally, studies indicate a possibly higher prescribing rate of antibiotics by remote consultations for urinary tract infections (UTIs) compared with in-person consultations.^19,20^ In Norway, it is not known to what extent acute cystitis is handled by remote consultations and whether resulting antibiotic treatment differs from in-person consultations. Cystitis is a high-incident condition, which entails that even small differences in doctor-seeking behaviour, form of contact, repeat contact, and antibiotic treatment could impact total antibiotic consumption, which is continually relevant in the ongoing antimicrobial resistance crisis.

Our hypotheses were H_0_: the mode of index consultation for acute cystitis had no effect on antibiotic treatment and repeat contacts versus H_a_: the mode of index consultation for acute cystitis affected antibiotic treatment and repeat contacts. The aim of this study was to examine whether there were differences in antibiotic treatment or repeat contacts for acute cystitis between remote and in-person consultations.

## Method

### Design and setting

This is an observational retrospective cohort study based on nationwide registry data from the publicly funded primary care in Norway. The Norwegian patient-list system entitles all residents to a named GP and 99.8% of the population has a RGP.^21^

### Data sources

Data from two national health registries for the complete 4-year period from 2018–2021 were linked at the individual level, using the unique personal identity number (encrypted) assigned to all residents of Norway.

The Control and Payment of Health Reimbursement (KUHR) Database contains data from all claims for fee-for-service from GPs and out-of-hours (OOH) services.^22^ For each contact, the claims contain GP and patient identifiers, date of contact, and one or more diagnoses, according to the International Classification of Primary Care, 2^nd^ edition (ICPC-2). KUHR does not include information from privately funded primary care services.

The Norwegian Prescription Database (NorPD) contains information on all prescription drugs dispensed from pharmacies to individual patients.^23^ For each prescription, NorPD contains encrypted prescriber and patient identifiers, date of dispensing, and generic drug information (Anatomical Therapeutic Chemical [ATC] code). The NorPD does not include information on medication administered to hospitalised patients or nursing home residents.

### Study population

The study population was women aged ≥16 years consulting with RGPs and OOH services for cystitis and pyelonephritis in Norway in the 4-year period from January 2018 until December 2021.

### Analytic units and variables

Acute cystitis episodes were constructed to identify and compare index consultations, using a 14-day UTI-related (diagnostic codes U71 cystitis and U70 pyelonephritis) contact-free and systemic antibiotics-free interval as washout. Exposure was the type of index consultation (either remote or in-person), marking the start of the acute cystitis episode. Outcomes were antibiotic treatment within 3 days and repeat contact within 14 days.

We defined the following three types of contacts: in-person consultations; remote consultations; and simple contacts. The types of contacts were based on reimbursement codes in KUHR and the definitions for these codes used by the Norwegian Institute of Public Health.^24^ Consultations (in-person or remote) involve a direct exchange of information between the patient and evaluation by the physician. Simple contacts have no direct communication between patient and physician but involve auxiliary personnel or are limited to simple one-way advice from the physician to the patient. The start of acute cystitis episodes was established by an index consultation (either in-person or remote) for U71 with a preceding period of at least 2 weeks without a contact for U71 or U70 or dispensing of any antibiotics for systemic use (ATC code J01). The first subsequent contact for either U71 or U70, within 2 weeks following the index consultation for U71, were considered as repeat contact; any later contacts within the 2 weeks following the index consultation, or before a new 2-week washout period, were disregarded, thus dichotomising the outcome of repeat contacts. Consultations referred to in this text are index consultations and confidence intervals 95% unless otherwise specified.

**Figure 1.**
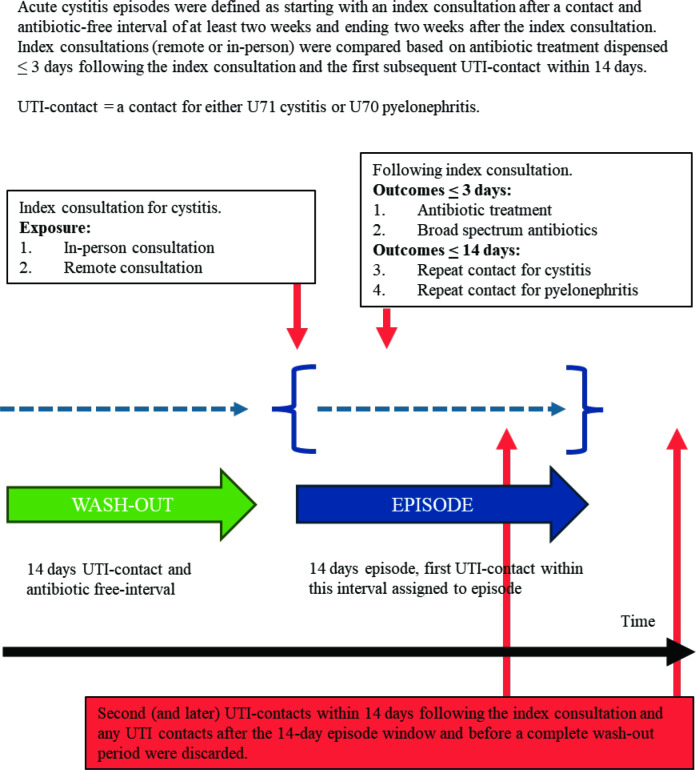
Construction of acute cystitis episodes in Norwegian primary care, 2018-2021

From NorPD we included all dispensed antibiotics for systemic use (ATC code J01) within 3 days following a KUHR registered consultation for U71 (Figure 1). Antibiotics were defined as first-line if among the three first choices for acute cystitis in Norwegian national guidelines, namely pivmecillinam, nitrofurantoin, or trimethoprim.^25^ Other antibiotics were defined as second-line.

Adjustment variables were selected by clinical judgement and subsequent univariate analyses with a significance cutoff of *P*<0.10.^26^ We adjusted for patient age, year, multimorbidity, pregnancy, and history of recurrent cystitis. For the outcome of repeat contact within 14 days, we adjusted for whether the preceding index consultation had resulted in antibiotic treatment within 3 days (Supplementary Table S1).

Multimorbidity was defined using the validated ICPC morbidity index introduced by Sandvik *et al* in 2022.^27^ The morbidity index measures the burden of health problems by chronic conditions and performs similarly to the Charlson Comorbidity Index in predicting mortality.^27^ We adopted the ICPC morbidity index to our dataset with the following time criterium: ICPC-2-code(s) recorded in the calendar year of the consultation (SupplementaryTable S2).

Pregnancy was defined as present if certain ICPC-2 diagnosis indicating pregnancy were registered within the preceding 6 months of the index consultation, with the simultaneous absence of diagnostic codes indicating a termination of pregnancy (Table 1). The interval of 6 months was decided as a balanced approach to avoid under- and over-estimation of concurrent pregnancies.

**Table 1.** ICPC-diagnostic codes used to define pregnancy

Codes indicating pregnancy if registered within 6 months preceding the index cystitis consultation	ICPC-2 codes
Antepartum bleeding	W03
Pregnancy vomiting/nausea	W05
Concern body image in pregnancy	W21
Limited function/disability	W28
Pregnancy symptom/complaint	W29
Infection complicating pregnancy	W71
Malignant neoplasm related to pregnancy	W72
Benign/unspecified neoplasm/pregnancy	W73
Injury complicating pregnancy	W75
Congenital anomaly complicating pregnancy	W76
Pregnancy	W78
Unwanted pregnancy	W79
Toxaemia of pregnancy	W81
Pregnancy high risk	W84
Gestational diabetes	W85
**With the simultaneous absence of the following codes:**	
Abortion spontaneous	W82
Abortion induced	W83

### Statistical analysis

Associations between mode of index consultation and antibiotic treatment and repeat contacts were examined by multivariate binomial regression analyses with an alpha (α) level of *P*<0.05. Analyses were performed using Stata SE for Windows (version 18.0). Tables and graphics were prepared with Microsoft Excel 2010.

## Results

### Study population, acute cystitis contacts and episodes

There were altogether 723 234 contacts for acute cystitis making up 586 371 episodes during the 4-year study period. Our dataset included 338 327 unique female patients.

Annual average number of acute cystitis contacts for the female population of Norway decreased slightly during the COVID-19 pandemic: from an annual average of 182 056 contacts (84.7 per 1000 inhabitants per year) for the years 2018 and 2019 to 179 561 contacts (82.2 per 1000 inhabitants per year) for the years 2020 and 2021 (Table 2). For the entire 4-year period, in-person consultations numbered 598 339 (82.7%), remote consultations 51 325 (7.1%), and simple contacts 73 570 (10.2%). Remote consultations for acute cystitis increased markedly, from 0.5% of acute cystitis consultations in 2018 to 15.2% in 2021. The mode of consultation described below are index consultations.

**Table 2.** Acute cystitis contacts (index consultations and first repeat contacts <14 days), women aged ≥16 years in Norwegian primary care, 2018–2021

Years	Inhabitants in norway	Number of cystitis contacts	U71 contacts in total per 1000 females	In-person consultations per 1000 females	Remote consultations per 1000 females	Simple contacts** per 1000 females
2018	2 139 995	177 498	82.9	73.9	0.4	8.6
2019	2 157 623	186 614	86.5	76.6	0.9	9
2020	2 177 584	181 398	83.3	64.7	10	8.6
2021	2 192 842	177 724	81	61.1	12.2	7.7
Annual average before COVID-19 (2018–2019)		182 056	84.7	75.2	0.6	8.8
Annual average during COVID- 19 (2020– 2021)		179 561	82.2	62.9	11.1	8.2
**Index consultations for acute cystitis resulting in antibiotic treatment within three days per 1000 inhabitants, women aged ≥16 and up years in Norwegian primary care, 2018–2021**						
**Years**	**Inhabitants in Norway**	**Number of index cystitis consultations resulting in antibiotics**	**All acute index cystitis consultations resulting in antibiotics per 1000 females**	**In-person consultations resulting in antibiotics per 1000 females**	**Remote consultations resulting in antibiotics per 1000 females**	
2018	2 13 995	115 453	54	53.7	0.2	
2019	2 157 623	121 526	56.3	55.9	0.4	
2020	2 177 584	117 516	54	47.5	6.4	
2021	2 192 842	115 213	52.5	44.6	7.9	
Annual average before COVID-19 (2018–2019)		118 490	55.1	54.8	0.3	
Annual average during COVID- 19 (2020–2021)		116 365	53.3	46.1	7.2	
**Repeat contacts for acute cystitis resulting in antibiotic treatment within three days per 1000 inhabitants, women aged ≥16 and up in Norwegian primary care, 2018–2021**						
**Years**	**Inhabitants in Norway**	**Number of repeat cystitis consultations resulting in antibiotics**	**All acute repeat cystitis consultations resulting in antibiotics per 1000 females**	**In-person consultations resulting in antibiotics per 1000 females**	**Remote consultations resulting in antibiotics per 1000 females**	**Simple contacts resulting in antibiotics per 1000 females**
2018	2 13 995	27 903	13	5.9	0.1	7.1
2019	2 157 623	29 898	13.9	6.2	0.2	7.5
2020	2 177 584	30 290	13.9	5.3	1.6	7.1
2021	2 192 842	28 540	13	4.8	1.9	6.3
Annual average before COVID-19 (2018–2019)		28 901	13.4	6.1	0.1	7.3
Annual average during COVID- 19 (2020– 2021)		29 415	13.5	5	1.7	6.7

*Limited to females aged ≥16 years. **Limited to repeat simple contacts, included if occurring <14 days following an index consultation.

The mean age for in-person consultations was 53.4 years (standard deviation [SD] = 21.7 years) and 52.9 years (SD = 20.7 years) for remote consultations. Proportionally, patients using remote consultations were more often pregnant or had multimorbidity; 5.7% and 16.9%, respectively, than patients using in-person consultations; 3.9% and 13.8%, respectively (Table 3).

**Table 3.** Index in-person versus index remote consultations for acute cystitis, Norwegian primary care, 2018–2021. Distribution of consultations by patient age groups, year of consultation, pregnancy, multimorbidity status, antibiotic treatment <3 days, and repeat contacts <14 days

	In-person consultation	Remote consultation	Total	χ2 test, P value
***n***	544 452 (92.9%)	41 919 (7.1%)	586 371 (100.0%)	
**Age groups, years**				
16-25	77 706 (14.3%)	4531 (10.8%)	82 237 (14.0%)	<0.001
26-35	70 625 (13.0%)	6532 (15.6%)	77 157 (13.2%)	
36-45	58 752 (10.8%)	5300 (12.6%)	64 052 (10.9%)	
46-55	68 024 (12.5%)	6188 (14.8%)	74 212 (12.7%)	
56-65	74 158 (13.6%)	5973 (14.2%)	80 131 (13.7%)	
66-75	94 846 (17.4%)	6256 (14.9%)	101 102 (17.2%)	
76-85	72 064 (13.2%)	4898 (11.7%)	76 962 (13.1%)	
86-95	26 745 (4.9%)	2098 (5.0%)	28 843 (4.9%)	
≥96	1532 (0.3%)	143 (0.3%)	1675 (0.3%)	
**Year**				
2018	144 064 (26.5%)	744 (1.8%)	144 808 (24.7%)	<0.001
2019	150 296 (27.6%)	1429 (3.4%)	151 725 (25.9%)	
2020	128 016 (23.5%)	17 803 (42.5%)	145 819 (24.9%)	
2021	122 076 (22.4%)	21 943 (52.3%)	144 019 (24.6%)	
**Pregnancy registered within the calendar year of the index consultation**				
Not pregnant	523 342 (96.1%)	39 545 (94.3%)	562 887 (96.0%)	<0.001
Pregnant	21 110 (3.9%)	2374 (5.7%)	23 484 (4.0%)	
**Multimorbidity registered within the calendar year of the index consultation**				
No other morbidities	469 432 (86.2%)	34 838 (83.1%)	504 270 (86.0%)	<0.001
Other morbidity	75 020 (13.8%)	7081 (16.9%)	82 101 (14.0%)	
**Recurrent cystitis in the calendar year of the acute cystitis episode**				
<3 cystitis episodes per year	511 394 (93.9%)	38 745 (92.4%)	550 139 (93.8%)	<0.001
>3 cystitis episodes per year	33 058 (6.1%)	3174 (7.6%)	36 232 (6.2%)	
**Antibiotics or not dispensed <3 days following index consultation**				
No antibiotic treatment	114 845 (21.1%)	9750 (23.3%)	124 595 (21.2%)	<0.001
Antibiotic treatment	429 607 (78.9%)	32 169 (76.7%)	461 776 (78.8%)	
**Types of antibiotics dispensed <3 days following index consultation**				
First-line	299 961 (69.8%)	21 987 (68.3%)	321 948 (69.7%)	<0.001
Second-line	129 646 (30.2%)	10 182 (31.7%)	139 828 (30.3%)	
**Repeat contact <14 days following index-consultation**				
No repeat contact	418 374 (76.8%)	31 134 (74.3%)	449 508 (76.7%)	<0.001
Repeat contact	126 078 (23.2%)	10 785 (25.7%)	136 863 (23.3%)	
**Type of repeat contact <14 days following index-consultation**				
In-person consultation	51 232 (40.6%)	2655 (24.6%)	53 887 (39.4%)	<0.001
Remote consultation	6273 (5.0%)	3133 (29.0%)	9 406 (6.9%)	
Simple contact	68 573 (54.4%)	4997 (46.3%)	73 570 (53.8%)	
**Antibiotics or not dispensed <3 days following repeat contact**				
No antibiotic treatment	71 693 (56.9%)	5959 (55.3%)	77 652 (56.7%)	<0.001
Antibiotic treatment	54 385 (43.1%)	4826 (44.7%)	59 211 (43.3%)	
**If antibiotic treatment <3 days following index consultation and <3 days following repeat contact, antibiotic switch?**				
Same antibiotic repeated	33 284 (26.4%)	3910 (36.3%)	37 194 (27.2%)	<0.001
Different antibiotic	92 794 (73.6%)	6875 (63.7%)	99 669 (72.8%)	
**Pyelonephritis contact <14 days following index consultation**				
Not pyelonephritis	123 823 (98.2%)	10 679 (99.0%)	134 502 (98.3%)	<0.001
Pyelonephritis	2255 (1.8%)	106 (1.0%)	2 361 (1.7%)	

### Antibiotic treatment within 3 days

Antibiotic treatment was dispensed more often following in-person consultations; 78.9% (95% confidence interval [CI] = 78.8% to 79.0%) than following remote consultations; 76.7% (95% CI = 76.3% to 77.1%). Second-line antibiotics were less often dispensed following in-person consultations; 30.2% (95% CI = 30.0% to 30.3%), than following remote consultations; 31.7% (95% CI = 31.1% to 32.2%).

Using in-person consultations as reference, remote consultations were associated with less antibiotic treatment (adjusted relative risk [aRR] 0.98, 95% CI = 0.97 to 0.98, *P*<0.001) but more second-line antibiotic treatment (aRR 1.04, 95% CI = 1.02 to 1.06, *P*<0.001) (Table 4).

**Table 4.** Association between type of index consultation for acute cystitis and antibiotic treatment (within 3 days) and repeat contacts (within 14 days), 2018–2021, Norwegian primary care. Binomial regression.

Unadjusted		Risk ratio	95% CI	*P* value (*α* = 0.05)
**1. Antibiotic treatment (any) within 3 days**				
In-person consultation		1		
Remote consultation		0.97	0.97 to 0.98	<0.001
**2. Second-line antibiotic treatment within 3 days**				
In-person consultation		1		
Remote consultation		1.05	1.03 to 1.07	<0.001
**3. Repeat cystitis contact within 14 days**				
In-person consultation		1		
Remote consultation		1.11	1.09 to 1.13	<0.001
**4. Repeat contact for pyelonephritis within 14 days**				
In-person consultation		1		
Remote consultation		0.55	0.45 to 0.67	<0.001
Adjusted* for:	**Outcomes and type of index consultation**	**Risk ratio**	**95% CI**	***P*** **value (α = 0.05)**
**Patient age, period, pregnancy, morbidity, and history of recurrent cystitis**	1. Antibiotic treatment (any) within 3 days			
	In-person consultation	1		
	Remote consultation	0.98	0.97 to 0.98	<0.001
**Patient age, morbidity, and history of recurrent cystitis**	2. Second-line antibiotic treatment within 3 days			
	In-person consultation	1		
	Remote consultation	1.04	1.02 to 1.06	<0.001
**Patient age, period, pregnancy, morbidity, history of recurrent cystitis and index antibiotic treatment**	3. Repeat cystitis contact within 14 days			
	In-person consultation	1		
	Remote consultation	1.11	1.09 to 1.12	<0.001
**Patient age, pregnancy, morbidity, history of recurrent cystitis and index antibiotic treatment**	4. Repeat contact for pyelonephritis within 14 days			
	In-person consultation	1		
	Remote consultation	0.57	0.47 to 0.70	<0.001

*Adjustments included were judged by the authors to be clinically relevant and met the cutoff point of *P*<0.10 by univariate analyses (χ2 test).

### Repeat contact within 14 days

Repeat contacts for cystitis occurred less often following in-person consultations; 23.2% (95% CI = 23.0% to 23.3%) than following remote consultations; 25.7% (95% CI = 25.3% to 26.1%).

The mean time to repeat contact following in-person consultations was 5.2 days (SD = 3.7) and for remote consultations 4.4 days (SD = 3.9). Acute pyelonephritis was observed more often following in-person consultations; 1.8% (95% CI = 1.7% to 1.9%) than following remote consultations; 1.0% (95% CI = 0.8% to 1.2%).

Using in-person consultations as reference, remote consultations were associated with more repeat contact for cystitis (aRR 1.11, 95% CI = 1.09 to 1.12, *P*<0.001), but less associated with a repeat contact for pyelonephritis (aRR 0.57, 95% CI = 0.47 to 0.70, *P*<0.001) (Table 4). Further details on repeat contacts are available in Supplementary Table S4.

## Discussion

### Summary

Remote consultations for acute cystitis increased markedly during the COVID-19 pandemic, with a simultaneous reduction in the annual total acute cystitis contacts.

Antibiotic treatment within 3 days was less common following remote consultations for cystitis, compared with in-person consultations. Second-line treatment was more common following remote consultations. Repeat contact for cystitis within 14 days was also more common following remote consultations.

### Strengths and limitations

All activity by GPs and OOH services must be reported to KUHR Database for reimbursements, ensuring a rich and complete dataset.^28^ Registry data still has inherent limitations that are important to be aware of when interpreting our findings. Diagnoses and reimbursement codes can deviate from what occurred in the consultation. The rate of some events may therefore be underestimated, and the rate of others overestimated.

Biases are reduced when using routinely and systematically collected data, although we as researchers might introduce systematic errors when we define variables such as illness episode duration, and chronic morbidities, besides the present acute UTI. It is difficult to assess the direction and magnitude of these biases. A longer episode duration definition would risk grouping separate episodes into one. A shorter duration would potentially underestimate the number of repeat contacts and thereby lead to loss of information on the tail-end of the illness episodes. We believe the effect of these limitations on the observed total trends and correlations in our results to be comparatively minor, considering that we are using a complete national dataset.

### Comparison with existing literature

A Dutch study by Nielen *et al* on defining illness episodes from registry data is relevant to our endeavour to define acute cystitis episodes to identify and compare modes of index consultations (remote versus in-person).^29^ In that study, it was suggested to use an 8-week contact and antibiotic-free interval for cystitis and half of this interval (4 weeks) as episode duration. We tried to make this suggested model work with our individually linked national and complete dataset but found that using different intervals for washout and episode duration led to episodes erroneously overlapping. Numerous studies have documented that uncomplicated cystitis, the most common presentation of cystitis, is expected to resolve within 1 week.^1,4^ Ultimately, we chose to construct our own episode definition for acute cystitis: washout of a minimum 2-week UTI contact and systemic antibiotic-free interval, episode start marked by an index consultation for cystitis and then a two-week episode duration.

The current understanding of the development of cystitis is that the ascending route of uropathogens is via the urethra into the bladder.^30^ One would then not necessarily expect an airborne pandemic and associated infection control measures to affect the incidence of UTIs. Among common infections during the COVID-19 pandemic, the incidence of UTIs was observed to be uniquely stable in one study from England, while the incidence of all other investigated infectious diseases fell.^31^ We found an overall decrease in the annual consultations for acute cystitis during the COVID-19 pandemic. Patients’ physician-seeking behaviour changed during the pandemic,^32^ and more patients with acute cystitis may have chosen a wait-and-see approach. A well-established risk factor for acute cystitis is sexual intercourse.^33^ A systematic review on the sexual health of women from 18 countries during the COVID-19 pandemic found a reduced frequency of sexual intercourse during the pandemic.^34^ If there was a similar trend in Norway this could contribute to the observed annual decrease of cystitis consultations in our study.

Several previous articles report higher antibiotic treatment rates for remote than for in-person consultations for infections.^19,20,35^ Conversely, we found that remote consultations for cystitis during the pandemic led to less antibiotic treatment overall within 3 days, but more repeat contacts during the subsequent 14 days. More repeat contacts could be interpreted in two ways; either the threshold for a repeat contact to the GP is lower following a remote consultation, or the acute cystitis was not adequately treated. The average time to repeat contact following index remote consultations was 4.4 days (SD = 3.9), which is after the time for the onset of maximum effect of the index antibiotic therapy (3 days) and repeat contacts could therefore either be an expression of therapy failure or a check-up to see if the infection had resolved. When antibiotics were dispensed following a remote consultation, they were more often second-line.

Repeat contact occurred in 23.3% of all acute cystitis episodes (Table 3), with 43.3% of these repeat contacts leading to antibiotic treatment, a finding that implies that in our study around 10% or less of acute cystitis episodes required repeat antibiotic treatment. This is similar to findings in the registry study from Denmark that found repeat antibiotic treatment in<10% of uncomplicated cystitis episodes.^5^ We did, however, not only examine uncomplicated cystitis, but also the acute cystitis episodes in our study included pregnant women, women with multimorbidity, and older people, which were adjusted for in the analyses.

Our findings show that increased use of remote consultations for acute cystitis in Norwegian primary care during the pandemic led to less initial antibiotic treatment. Contributing to this observation could be that Norwegian primary care is organised as a publicly funded RGP-scheme and has well-defined antibiotic guidelines, which in general advise against antibiotic treatment via remote consultations.^25^ Another important factor is the continuity of the patient–physician relationship following the RGP scheme, which has proven to reduce the use of OOH services, acute hospital admissions, and mortality.^36^ Continuity in the patient–physician relationship has also been suggested to reduce inappropriate antibiotic prescriptions.^37^ The choice of consultation modality is at the discretion of the patient, which highlights the need for increased patient guidance, to ensure safe use of remote consultations.^12^ More second-line treatment following index remote consultations could be an expression of uncertainty about the severity of the UTI in question on the part of the GP, leading to prescription of what could be perceived as more effective treatment, second-line antibiotics, just in case. Repeat contacts after index remote consultations resulted in proportionally more antibiotics than repeat contacts following in-person consultations (44.7% versus 43.1%, *P*<0.001) (Table 3). Cystitis episodes initiated by remote consultations and followed up with secondary remote consultations were characterised by a comparable proportion of antibiotic treatment overall, but more repeat contacts and more second-line antibiotics.

In our study, repeat contacts for pyelonephritis within 14 days occurred in 1.7% of all acute cystitis episodes, in line with the registry study from Sweden, which found that about 1% of untreated uncomplicated cystitis episodes were followed by pyelonephritis within 30 days.^6^ We found a comparatively stronger association between an in-person consultation and subsequent contact for pyelonephritis. We interpret this as possible selection bias; patients who had the early hallmarks of pyelonephritis may have preferred in-person consultation rather than to be evaluated by remote consultation. Another possibility is that the threshold for seeking an in-person consultation could be higher than for a remote consultation, which introduces a delay in the index contact for acute cystitis. Aligning with this view is that repeat contacts occurred later following in-person (mean = 5.2 days, SD = 3.7), than remote consultations (mean = 4.4 days, SD = 3.9 days) and that when a repeat contact occurred, index remote consultations were more often followed by a remote consultation, compared with index in-person consultations (29.0% versus 5.0%, respectively, Table 4). Acute cystitis itself occurs *before* a patient meets with the physician and our earliest datapoint is the index contact, when the patient already has had an infection or infectious-related symptoms for some time.

The acute cystitis episodes in this study were not strictly compatible with the classic definition of uncomplicated acute cystitis; for instance, we chose to include all females aged ≥16 years and included pregnancy as an adjustment variable, rather than being an exclusion criterion. The age of majority in matters of health is 16 years in Norway and this is the earliest age patients can use remote consultations by themselves, which marked a natural inclusion point for our study, comparing remote and in-person consultations. Defining pregnancy in registry-based studies is challenging but important, as acute cystitis frequently occurs in women of childbearing age. Acute cystitis in pregnant women is, however, not uncomplicated and Norwegian guidelines recommend prompt antibiotic treatment.^25^ A Norwegian study combined data from three national registries for the years 2009–2013 and found 421 201 pregnancies in total or around 84 240 pregnancies per year.^38^ This divided by the average annual Norwegian female population aged ≥16 years during the same period (1 979 333 women), as included in our study,^39^ gives around 4.3% of Norwegian women being pregnant, per year. Our definition of pregnancy (Table 1) arguably gave a reasonable estimate of 4.0% of women with acute cystitis aged ≥16 years being labelled as pregnant (Table 3).

A slightly higher proportion of patients using remote consultations were pregnant or had multimorbidity (5.7% and 16.9%), compared with (3.9% and 13.8%, respectively) for in-person consultations. One possible explanation could be an increased worry for contracting COVID-19 during the height of the pandemic in these patient groups or perhaps the convenience of remote consultations, or both. These findings underscore how remote consultations can expand access to care.

### Implications for research and practice

Our findings are influenced by the specific conditions in the Norwegian primary care system and the unique period the COVID-19 pandemic represented. A relatively low rate of antimicrobial resistance and the Norwegian RGP scheme are two challenges to the generalisability of our findings to primary care in other countries. Further validation of our suggested method to define pregnancy in ICPC2 registry-based studies (Table 1) could be an interesting avenue for future research.

The increased use of remote consultations has endured in the years following the pandemic.^40^ The Norwegian Directorate of Health now aims to establish an online GP service and to mandate by law that RGPs offer remote consultations.^18^ Remote consultations can expand access to care, but our findings suggest some important limitations that policymakers should be aware of, including the observed increased dispensation of second-line antibiotic treatment.

## Data

Availability of data and materials: The data that support the findings of this study are available from The Control and Payment of Health Reimbursement (KUHR) database and The Norwegian Prescription Database (NorPD), but restrictions apply to the availability for these data, which were used under license for the current study, and therefore are not publicly available. However, data are available from the authors upon reasonable request and provided granted permission from the KUHR database, NorPD and REC West Norway.
